# Diagnostic Biomarkers for Gestational Diabetes Mellitus Using Spectroscopy Techniques: A Systematic Review

**DOI:** 10.3390/diseases11010016

**Published:** 2023-01-25

**Authors:** Rabia Sannam Khan, Haroon Malik

**Affiliations:** 1 Department of Bioengineering, Lancaster University, Lancaster LA1 4YW, UK; 2 Queens Medical Centre, Jumeirah, Dubai P.O. Box 2652, United Arab Emirates

**Keywords:** gestational diabetes mellitus, biomarker, biofluids, spectroscopy, prediction, diagnosis

## Abstract

Gestational diabetes mellitus (GDM) is associated with adverse maternal and foetal consequences, along with the subsequent risk of type 2 diabetes mellitus (T2DM) and several other diseases. Due to early risk stratification in the prevention of progression of GDM, improvements in biomarker determination for GDM diagnosis will enhance the optimization of both maternal and foetal health. Spectroscopy techniques are being used in an increasing number of applications in medicine for investigating biochemical pathways and the identification of key biomarkers associated with the pathogenesis of GDM. The significance of spectroscopy promises the molecular information without the need for special stains and dyes; therefore, it speeds up and simplifies the necessary ex vivo and in vivo analysis for interventions in healthcare. All the selected studies showed that spectroscopy techniques were effective in the identification of biomarkers through specific biofluids. Existing GDM prediction and diagnosis through spectroscopy techniques presented invariable findings. Further studies are required in larger, ethnically diverse populations. This systematic review provides the up-to-date state of research on biomarkers in GDM, which were identified via various spectroscopy techniques, and a discussion of the clinical significance of these biomarkers in the prediction, diagnosis, and management of GDM.

## 1. Introduction

Gestational diabetes mellitus (GDM) is a carbohydrate intolerance resulting in hyperglycaemia of variable severity with the onset or first recognition during pregnancy [1]. As GDM is glucose intolerance of a severe degree, which is usually diagnosed in any trimester of pregnancy, it exerts maternal and neonatal risks [2]. GDM itself is the most common complication of pregnancy, and has increased more than 30% in the past two decades in many developing countries [3]. GDM occurs in more than 10% of pregnancies and increases the risk of complications of pregnancy, including preeclampsia, placental abnormalities, stillbirth, emergency caesarean, and future development of type 2 diabetes mellitus (T2DM) in children and in mothers [4].

Moreover, GDM imposes a seven-fold increased risk of T2DM in women in later life [5]. The increase in risk factors such as overweight, sedentary life style, unhealthy diet, and diabetes prevalence rate has risen quicker in low- and middle-income countries as compared to high-income countries [6]. Globally, 422 million adults had diabetes in 2014, in contrast to 108 million in 1980, which illustrates the quadrupled global prevalence of diabetes since 1980. It has risen from 4.7% to 8.5% in the adult population. Overall, depending upon the diagnosis criteria applied, the country-specific prevalence of GDM estimation available reveals that North Africa and the Middle East have the highest prevalence of GDM at 12.9% [7,8], followed by Southeast Asia at 11.7%, the Western Pacific at 11.7%, South and Central America and Africa at 11.2%, North America at 8.9%, and the Caribbean at 7.0%. However, Europe had the lowest prevalence rate, at 5.8% [9].

Diagnostic criteria and common screening approaches have varied among different countries in different periods of time. There have been ongoing debates on the optimum approach/method and the diagnostic criteria have evolved rapidly since 1964, from the Somogyi–Nelson technique for GDM diagnosis [10] up to the recently used World Health Organization (WHO) (2013 to present) diagnostic criteria. The most common diagnostic criteria include those of the WHO, American Congress of Obstetricians and Gynecologists, Canadian Diabetes Association, and International Association of Diabetes and Pregnancy Study Groups (IADPSG) [11]. The WHO and IADPSG criteria still remain the communal universal screening criteria for all pregnant women, in which gestational diabetes is determined by a standard 75 g oral glucose tolerance test (OGTT) performed after overnight fasting (8–14 h) in 250–300 mL water between the 24th and 28th weeks of gestational age. Pregnant women who fit into the WHO criteria for diabetes mellitus or impaired glucose tolerance (IGT) are categorized as having GDM [1].

Given the accelerating burden of GDM worldwide, earlier identification of GDM is critical for prevention strategies. Prior efforts for identification of specific biomarkers are limited with the usage of different techniques. Spectroscopy techniques have emerged as potential tools in biomedical research, and in recent years, applications of these techniques have increased a great deal in the field of clinical assessment [12,13]. These techniques have not been much explored in the field of obstetrics and diabetes. There are several spectroscopic techniques employed in the clinical field, such as Raman spectroscopy (RS), Fourier Transform Infrared spectroscopy (FTIR), Elastic Scattering spectroscopy (ESS), Fluorescent spectroscopy (FS), and Nuclear Magnetic Resonance (NMR) spectroscopy [14,15,16,17,18,19,20,21,22,23,24,25,26]. The major advantages of these techniques over conventional imaging approaches are that they are less invasive, are reagent free, and allow in vivo measurements and ex vivo probes. Spectral data can be collected within seconds, which leads to quick detection and multidimensional data being collected from vital organs, where surgery is contraindicated [14,26,27].

Furthermore, advances in biphotonic engineering and data processing technology have led to the role of spectroscopic techniques in understanding human disease being widely expanded [28]. Spectroscopy is defined as the study of the interaction of electromagnetic radiation with atoms and molecules, which results in transitions in their energy state, i.e., from a stable to higher energy excited state. During this process, energy can be released, absorbed, scattered, or transformed. In addition, spectroscopic methods can be used to characterize biological samples in vitro and in vivo, along with the measurement of concentrations of compounds within the samples [29].

Hence, spectroscopic techniques can be used for the detection and development of biomarkers for diseases. Early detection of biomarkers will lead to improved interventions and consequently a reduction in mortality and morbidity [30]. Therefore, the aim of this systematic review is to evaluate primary research articles on the use of spectroscopy for prediction and diagnosis of GDM. Patterns of spectroscopic fingerprints found in the literature will direct future research and are discussed here as a potential diagnostic tool for GDM.

## 2. Materials and Methods

### 2.1. Focused Question

Based on The Preferred Reporting Items For Systematic Review And Meta-Analysis (PRISMA) guidelines [31], a specific question was constructed. The focused question was, “*Are spectroscopic techniques effective in the diagnosis of gestational diabetes mellitus*”?

### 2.2. Selection Criteria

The screening and assessment of articles were performed independently by two reviewers, R.K. and H.M. The following eligibility criteria were necessitated:

- Study design: Case–control and cohort studies from the journal articles were included.
- Participants: Including subjects with measures of GDM and/or controls. Studies included participants of age 18 years or above. Definition of GDM was based on WHO criteria or diagnosis by an obstetrician or endocrinologist based on IADPSG criteria.
- Language: Articles published only in English language.

In vitro studies, studies defining GDM without oral glucose tolerance test (OGTT), all other types of diabetes mellitus, i.e., diabetes mellitus type 1 and juvenile; review articles, case reports, cross-sectional studies, and animal studies were excluded.

### 2.3. Search Strategy

Two authors, R.K. and H.M., searched for all the published studies, including electronic searches and hand searching. Detailed search plans considered appropriate for each database were established. The reference lists of included studies were checked for other papers that might be suitable for inclusion. PubMed, MEDLINE, COCHRANE LIBRARY, SCOPUS, and CINAHL databases from start of March 2018 to May 2022 were searched for articles addressing the focused question. A logical and structured approach to literature searching was used for the identification of relevant papers that reported on spectroscopic techniques utilized in the diagnosis of biomarkers in GDM patients. For the PubMed library, combinations of the following MeSH (Medical Subject Headings) and free texts were used: (((“spectrum analysis”[MeSH Terms] OR (“spectrum”[All Fields] AND “analysis”[All Fields]) OR “spectrum analysis”[All Fields] OR “spectroscopy”[All Fields]) AND (“methods”[Subheading] OR “methods”[All Fields] OR “techniques”[All Fields] OR “methods”[MeSH Terms] OR “techniques”[All Fields])) OR ((“spectrum analysis”[MeSH Terms] OR (“spectrum”[All Fields] AND “analysis”[All Fields]) OR “spectrum analysis”[All Fields] OR “spectroscopy”[All Fields]) AND (“analysis”[Subheading] OR “analysis”[All Fields]))) AND (“diabetes, gestational”[MeSH Terms] OR (“diabetes”[All Fields] AND “gestational”[All Fields]) OR “gestational diabetes”[All Fields] OR (“gestational”[All Fields] AND “diabetes”[All Fields] AND “mellitus”[All Fields]) OR “gestational diabetes mellitus”[All Fields]).

### 2.4. Screening Methods and Data Abstraction

Titles and abstracts of articles which fulfilled the selection protocol were screened by 2 authors (R.K. and H.M.) and checked for settlement. The evidence from the accepted studies was formulated according to the (1) study design, (2) demographic characteristics of individuals, (3) age of individuals in study, (4) studies defining GDM with selected diagnostic criteria, (5) study outcomes, (6) type of spectroscopy techniques used, (7) types of biomarkers detected and analysed, (8) type of diabetes, (9) type of biofluid used for analysis, (10) sample size, and (11) main findings. Authors were contacted to obtain missing information and permission to reproduce their data where necessary.

Agreement between the 2 reviewers regarding the study selection process was planned, and one investigator performed the data extraction which was further confirmed by the secondary investigator.

### 2.5. Study Selection

**Figure 1:**
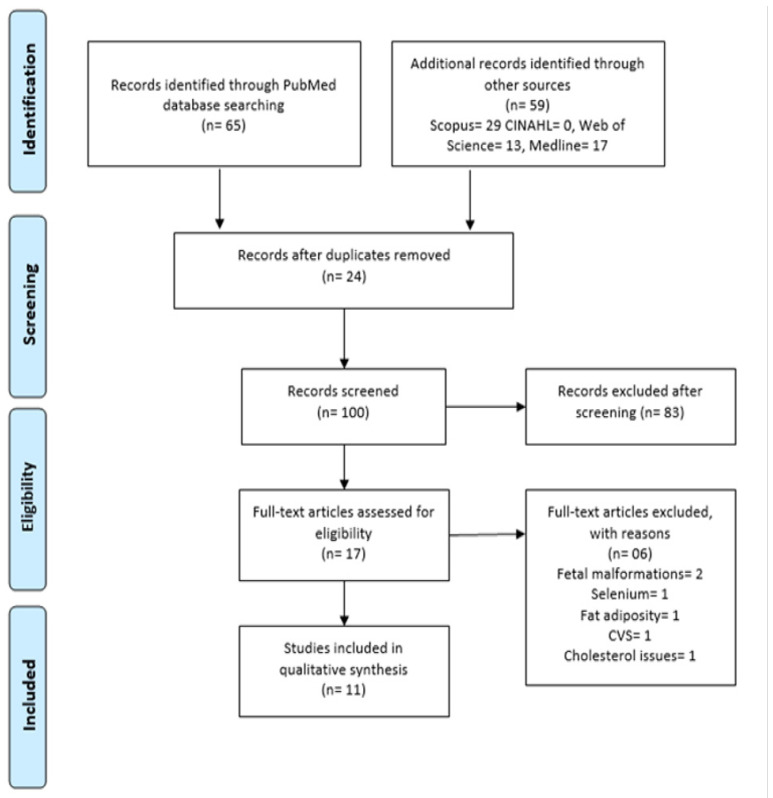
PRISMA Flow Chart for Studies Retrieved Via Searching and Selection Process.

The search protocol is presented in Figure 1. A total of 124 studies were primarily identified by following the PRISMA protocols. After screening of the titles and abstracts, 107 articles were excluded, and 17 articles were selected for detailed full-text screening. Out of these 17 studies, 6 studies were additionally excluded due to not fitting into eligibility criteria. After the final phase of selection, 11 case–control and cohort studies were included in this systematic review. All the studies were executed in hospitals or healthcare centres. Figure 1 shows the documentation in a flow chart with the reason for exclusion of articles.

### 2.6. Methodological Study Quality Assessment

Two authors, R.K. and H.M., independently assessed the methodological quality of included studies based on the revised recommendations of the Consolidated Standards of Reporting Trials statement (CONSORT) [32]. Studies included each have a clear hypothesis, aim, and outcome measures, as shown in the Table 1 below.

## 3. Results

### 3.1. Study Selection

#### General Characteristics of Included Studies

A total of 124 study titles and abstracts were primarily identified. After the removal of replica articles, one hundred and seven were excluded as inappropriate to the focused question or aim of the systematic review. A total of 17 were selected for full-text detailed reading. Of those 17 studies, 6 studies were further excluded. After the final stage of selection, 11 studies were included and administered for data extraction. Figure 1 shows the PRISMA flow chart with the reasons for exclusion of articles. The representation of the outcome of studies, specific biomarkers at different gestational time points, biological samples and the relevant spectroscopy techniques used, and a summary of the general characteristics and outcomes of the GDM selected studies can be seen in Table 2 and Table 3.

**Figure 2:**
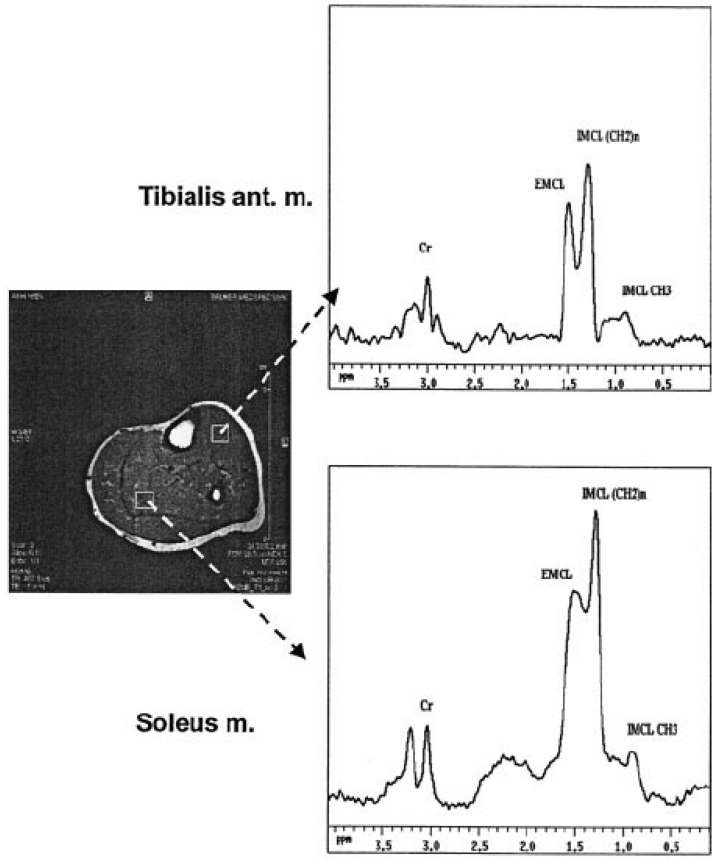
The cross-sectional image of human calf muscle via magnetic resonance imaging (**left side**). HNMR spectra attained from volumes of soleus and tibialis anterior muscles (**right side**) [33].

In Figure 2, Willer et al. explains how IMCL was measured through an NMR spectrometer, which was equipped with a gradient coil. On the right leg of the subject tibialis anterior muscles or soleus, the STEAM sequence which was complemented by CHESS water suppression was placed. By using the software MacNUTS-PPC, spectra were fitted and lines broadened [33].

**Figure 3:**
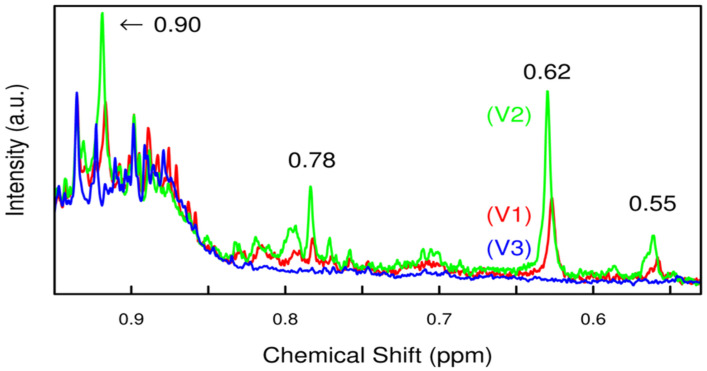
The four influential NMR signals over time [36].

In Figure 3, Sachse et al. shows the NMR spectra collected from three urine samples of healthy participants between the regions of 0.5 to 0.9 ppm, and creatinine concentration was normalized. The highlighted signals increase from V1 to V2 and disappear at V3. V1 is indicated with a red line and gestational weeks 8–20, V2 is indicated with a green line and gestational weeks 26–30, and V3 is indicated with blue line and 10–s16 weeks postpartum [36].

**Figure 4:**
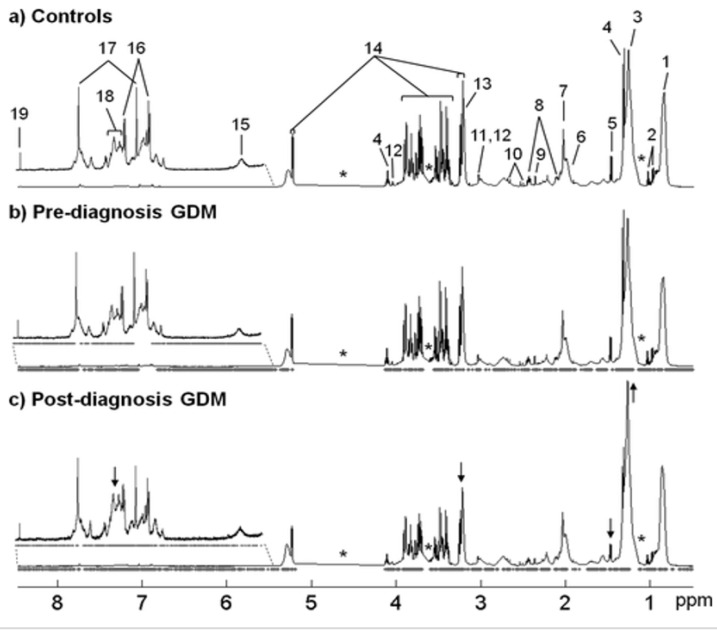
The HNMR spectra of plasma of (**a**) controls, (**b**) pre-diagnosis GDM, and (**c**) post-diagnosis GDM. Arrows specify the visible alterations; * = excluded spectral regions. (1) CH_3_ lipids, (2) Val, (3) (CH_2_)n lipids, (4) lactate, (5) Ala, (6) CH_2_C=C lipids, (7) N-acetyl glycoproteins, (8) Gln, (9) pyruvate, (10) citrate, (11) creatinine, (12) creatinine, (13) N(CH_3_)_3_ choline of PL, (14) glucose, (15) urea, (16) Tyr, (17) His, (18) unknown (δ 7.15–7.35), and (19) formate [39].

Pinto et al. shows in Figure 4 the selected data points in PLS-DA models. The changes can be seen in post-diagnosis GDM groups such as in lipids, choline of phospholipids, alanine, and unknown resonance. However, no obvious changes can be seen in pre-diagnosis GDM. Furthermore, there is a small increase in lipid resonances from controls to pre-diagnosis and ultimately to post-diagnosis GDM. Moreover, the spectra indicate and give the entire information related to components of plasma, lipoproteins, lipids, cholesterol, sphingomyelins, and phosphatidylcholines [39].

**Figure 5:**
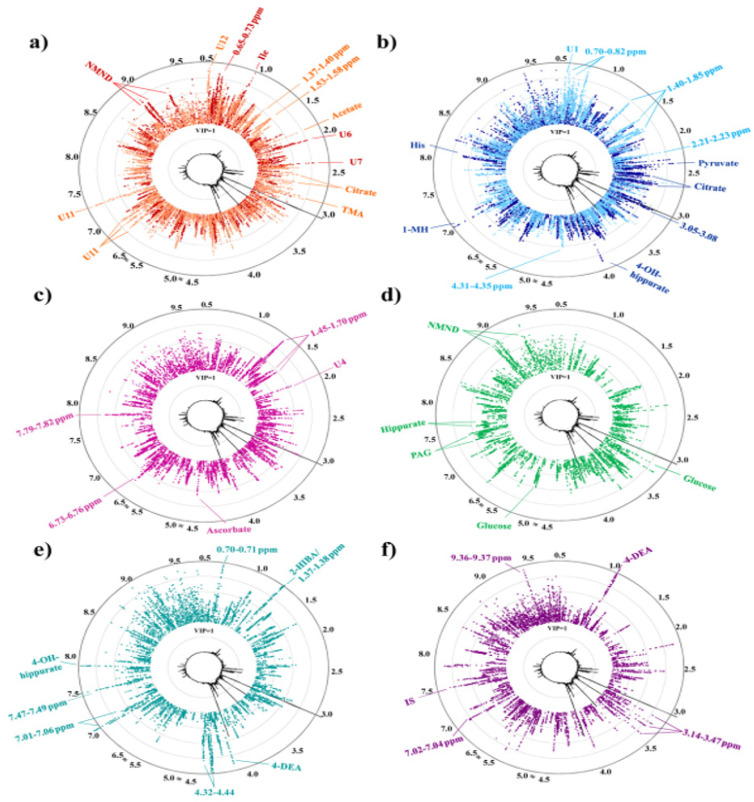
NMR metabolite signatures in VIP wheel presentation acquired for each prenatal disorder. Each dot represents a spectral data point. Average of HNMR spectrum of controls shown in inner circle with parts per million scale in outer black circle: (**a**) FM malformed foetuses = red, overlapped with CNS malformations = orange; (**b**) CD = dark blue, overlapped with T21= light blue; (**c**) pre-PTD = pink; (**d**) pre-diagnostic GDM = green; (**e**) pre-IUGR = turquoise; (**f**) pre-PE = purple. [44].

Diaz et al. in Figure 5 depicts the several variable importance to the projections (VIPs) and circular representations of the VIP wheel of urinary metabolic urinary signatures for women carrying foetuses with central nervous malformations, other foetal malformations, trisomy 21, and other chromosomal disorders [44]. This study demonstrated the value of maternal urine profiling, which is useful for prenatal diagnostics and early prediction of poor pregnancy outcomes.

## 4. Discussion

The current systematic review was based on the hypothesis that spectroscopic techniques can be used for the diagnosis of GDM through identification of biomarkers in biofluids of pregnant women. Overall, the studies included in the systematic review showed that spectroscopy techniques can be used for prediction and diagnosis of GDM. Biomarker research for GDM previously has been carried out through Enzyme-Linked Immunosorbent Assay (ELISA), Polymerase Chain Reaction (PCR), Reverse Transcriptase q Polymerase Chain Reaction (RT-q PCR), Western Blotting, and other molecular techniques [45]. Although these techniques are rapid and highly sensitive, they still have pitfalls, such as extensive sample collection protocols, sample selection criteria (best sample to be identified), handling conditions, and storage temperature (as minimal changes can lead to degradation of molecules); therefore, sample transportation to the laboratory plays an important role in the accuracy of results. Similar to other laboratory techniques, they can give false-positive and false-negative results, and contamination of samples is a major challenge, which can happen quickly with these techniques [46].

The need to develop and look for biomarkers is very much needed for the increasing rate of GDM, as the 75 g 2 h OGTT remains the only diagnostic test, which is cumbersome for the pregnant women [47,48,49]. Spectroscopy offers an excellent potential as a diagnostic tool, and biofluids can be analysed by using vibrational spectroscopic techniques. Therefore, it is essential to interpret the findings that were already reported in the literature, and this will help to develop further research in this discipline.

Prior to a deep dive in use of spectroscopy, it is important to understand what other techniques have been used to identify biomarkers. In a study reported by Kautzky-Willer et al., they measured pGDM (IMCL) with NMRS in tibialis anterior muscles (IMCL-T) and in the soleus. Glucose effectiveness (SG) and insulin sensitivity index (SI) were assessed from sampled glucose tolerance tests and body fat mass (BFM) from bioimpedance analysis. Therefore, IMCL-T reflected insulin sensitivity; however, IMCL-S was related to obesity. Their results showed that IMCL could serve as an additional parameter/biomarker for increased diabetes risk, since it identifies insulin resistance in pGDM patients and also those patients who were diagnosed previously and/or required insulin throughout pregnancy [33]. Furthermore, in a study conducted by Prikoszovich et al., they used MRS for the analysis of ectopic lipids (intramyocellular lipids (IMCL)) and the liver (hepatocellular lipids (HCL)) in glucose-tolerant non-obese pGDM with normal glucose metabolism women during pregnancy. They all underwent OGTT and intravenous glucose-tolerant tests 4–5 years after delivery. The results revealed that glucose-tolerant pGDM had high liver fat but slightly low muscular insulin sensitivity and ATP synthesis, suggesting that alteration in hepatic lipid storage signifies the early and predominant abnormality [34].

However, in 2012 the group of Graca et al. studies second trimester maternal urine and amniotic fluid by using UPLC-MS data comparable to NMR data. The aim was to investigate metabolic effects in GDM, foetal malformations (FM), and preterm delivery. The results showed the potential use of MS and NMR metabonomic studies for fully addressing the diseases, as they observed metabolic changes in the FM group with foetal hypoxia, hindered kidney development, and enhanced gluconeogenesis, while no changes were observed in the GDM and preterm delivery groups [50]. NMR metabolomics were used by the Sachse group for the identification of novel biomarkers in GDM. They collected maternal urine at three different time points of pregnancy, as mentioned above in the Table 2, from a multi-ethnic 823-person healthy cohort population. PCA, PLS-DA, and univariate statistics were also applied to see the differences. The experiment suggested that NMR techniques monitored changes in the urinary excretion profile of pregnant women [36]. In the same year, the other group aimed to evaluate the relationship between the pGDM fatty liver (FL) and future manifestation of T2DM. They used 1H-Magnetic Resonance Spectroscopy techniques and selected 68 pGDM patients and 29 controls at the experimental time of 3–6 months after delivery and assessed specific metabolic status. The results depicted that FL was associated with pGDM and insulin resistance and inflammation, and the potential risk of development of T2DM was higher in the pGDM group [35].

Diaz et al., in 2013, used HNMR metabolomics for analysis of second trimester maternal urine for the aim of early diagnosis of GDM, chromosomal disorders, foetal malformations, preterm delivery, preeclampsia, and intrauterine growth restriction. They were the first group to highlight the importance of profiling of maternal urine for the prediction of poor pregnancy outcomes and prenatal diagnosis [38]. In another study conducted by Pinto et al., they focused on the prediction of GDM through NMR metabolomics from maternal blood and corresponding lipid extracts. Metabolomic biomarkers from pre- and post-diagnosis GDM patients were sought between diseased and control patients through multivariate analysis of selected ^1^HNMR spectra. Variable selection of NMR spectra gave a classification model for pre- and post-diagnostic GDM. After GDM diagnosis, enhanced changes were seen along with low molecular compounds. They stated that via exploitation of multivariate profile changes, GDM prediction was possible. Furthermore, the 26-resonance plasma biomarker was successfully classified for rapid analysis of post-diagnosis GDM. The results of the study concluded that plasma as a biofluid was preferable for the detection of necessary biomarkers, and that NMR metabolomics can be used in GDM management [39]. In a cross-sectional study, 147 women were recruited to metabolically characterize women with recent GDM and a cohort at risk for T2DM. They used several methods such as insulin sensitivity index, disposition index, magnetic resonance imaging, and clinical chemistry. The conclusion of the study was that fetuin-A and leptin signalling were involved in the pathogenesis of T2DM identified as the early contributors for T2DM pathogenesis [41].

Pinto et al., in 2016, used urine NMR metabolomics that enabled a 12-resonance metabolic signature of GDM that could be identified at the time of diagnosis. This signature helped in the evaluation of the impact of insulin, responsive metabolic pathways, and in the identification of side effects [42]. Evidence suggests that NMR, MRS, and FTIR spectroscopy techniques were the most common techniques to be used for the analysis of biofluids in GDM.

Aydemir et al. reported on the relationships between maternal levels of lipid peroxidation marker malondialdehyde (MDA), oxidized LDL (ox-LDL), K167N single-nucleotide polymorphisms, and LOX-1 30UTR188C/T in 116 Turkish pregnant women with GDM. They were compared with 120 healthy pregnant women by using spectrophotometric methods and ELISA. Their results revealed that several environmental and genetic factors could be risk factors for GDM. The experiments revealed that the K167N polymorphism and LOX-1 30UTR188C/T are not involved in susceptibility to GDM, while both LOX-1 and various LOX-1 single-nucleotide polymorphisms need further evaluation. LOX-1 genetic polymorphisms could be related to decreased or increased ox-LDL and MDA levels and ultimately the risk of development of GDM. Hence, in their study MDA and ox-LDL levels were significantly high in GDM patients [51]. Moreover, in Turkish pregnant women, it was concluded that TT/NN genotype carriers were related to increased oxidative stress [40]. Recently, Jin et al. reported on a study to detect the difference in the metabolic profile in mild GDM by using ^1^HNMR spectroscopy and mRNA expression analysis. Thirty-six pregnant women with mild GDM and thirty-six with normal glucose tolerance (NGT) were selected. Their results clearly showed the understanding of underlying mechanisms of mild GDM by indicating the disturbance in glucose metabolism, amino acids, and fatty acid metabolism as well as the activate inflammatory response in GDM [43].

Biofluids, plasma, blood, urine, amniotic fluid, plasma lipid extracts, and the combination of biofluids were analysed by spectroscopic techniques such as ^1^HNMR, NMRS, MRS, and UPLC-MS in order to detect the GDM biomarkers. Based on the results, it was claimed that the ^1^HNMR spectroscopy technique with increased specificity and sensitivity was a good methodology to detect the changes and upregulation and downregulation of biomarkers in GDM patients [50,51].

Most of the studies focused on diagnostic and predictive biomarkers for GDM and on characterization of metabolic changes related to development of GDM. Biological samples of different forms were used with a diverse range of spectroscopic analytical platforms. Due to the advancement of new technologies, comprehensive models of care with benefits and long-term health outcomes for maternal and neonatal cases can be developed with the help of predictive and diagnostic biomarkers [52]. Since spectroscopy does not need any specialized reagents, and is easily adaptable, it can be used for the improved antenatal monitoring capacity, hence serving as a point of care in low-resource countries as well. Handheld devices such as for vibrational spectroscopy have already been in use for several years in the pharmaceutical industry, and their transition into the field of diagnostics has proven its high specificity and sensitivity. Therefore, the continued advances in handheld devices and the development of chemometric biomarkers would offer a low-cost viable predictor tool that will be used in the obstetric field successfully. Even though each spectroscopy technique has maximized its advantages, applications in sensing and imaging continue evolving to open new avenues of disease diagnosis, determination of unknown biological processes, and instrumentation. However, spectroscopy techniques do face challenges, as they need expertise in sample handling and processing, as results can be affected by even minute inaccuracy and can lead to misdiagnosis. Other limitations include the exact site to measure the glucose levels and the lack of clinical community engagement in spectroscopic advancements. Moreover, high-power lasers have the potential to burn or degrade samples, and therefore a precise tuneability of the lasers to have accurate sample exposure is essential. In addition, the accurate interpretation of spectral data by spectroscopists and its combination with chemometric analysis makes these methodologies more attractive.

## 5. Conclusions and Outlook

This systematic review provided an overview of the accelerating burden of GDM worldwide and the need for earlier identification of GDM for prevention strategies. Spectroscopic techniques can be used for the diagnosis of GDM through the identification of biomarkers in biofluids of pregnant women. A large number of trials with varied spectroscopy techniques in several populations with different types of biofluids were examined and the effectiveness of spectroscopy was assessed in order to identify biomarkers that can be used for diagnostic purposes. A number of studies focused on diagnostic and predictive biomarkers for GDM and on the characterization of metabolic changes related to the development of GDM. Vibrational spectroscopy can also be used for the diagnosis of GDM through the identification of biomarkers in biofluids of pregnant women. The next step should be to conduct research in larger, prospective, and more ethnically diverse populations before the biomarkers can be utilized in clinical practice. Furthermore, artificial intelligence (AI) and machine learning (ML) may be utilized in combination with FTIR and Raman spectroscopy (vibrational spectroscopy) in order to attain early diagnosis, and this is the direction towards a personalized medicinal approach.

## Figures and Tables

**Table 1:** Quality assessment of included studies by using the Newcastle–Ottawa Scale.

Country (First Author, Year)	Quality Indicators		
	Selection	Comparability	Exposure
Austria [33]	***	*	**
Austria [34]	**	*	***
Vienna [35]	***	*	**
Norway [36]	**	*	***
Portugal [37]	***	*	**
Portugal [38]	**	*	***
Portugal [39]	**	*	**
Turkey [40]	***	*	**
Germany [41]	***	*	**
Portugal [42]	**	*	***
China [43]	***	*	**

The stars (*) are representative of good, fair and poor quality of selection, comparability and outcome of studies 1 (*) is given for each numbered item of selection and comparability which includes (representative exposed cohort, ascertainment of exposure, and demonstration that outcome of interest was not present at start of study and comparability of cohorts on the basis of the design or analysis controlled for confounders). 2 stars (**) in selection domain AND 1 or 2 (**) in comparability domain AND 2 or 3 stars in outcome/exposure domain which indicates fair quality of study. 3 or 4 (***) in selection domain AND 1 or 2 stars in comparability domain AND 2 or 3 (***) in outcome/exposure domain represents the good quality of studies.

**Table 2:** The outcome of studies, specific biomarkers at different gestational time points, biological samples, and the relevant spectroscopy techniques used.

Investigators; Country	Study Design	Analytical Platform	Upregulated and Downregulated Biomarkers	Biological Samples	Gestational Time Point	Outcome of Study
Willer et al.; Austria [33]	Cohort	H NMRS	IMCL and raised plasma total leptin concentrations associated with insulin secretion, resistance, and BFM in pGDM	Blood, plasma	Pre-diagnosis GDM (2−21 gestational weeks prior to diagnosis) Post-diagnosis GDM (24−27) controls	The study showed that higher IMCL was related to risk factors for T2DM in the selected group of women and also in addition to metabolic syndrome, and it serves as a biomarker of risk for T2DM later in women with pGDM.
Prikoszovich et al.; Austria [34]	Cohort	Magnetic resonance spectroscopy	IMCL and HCL were high in pGDM	Plasma glucose	23 pGDM and 8 women without any risk factors for T2DM served as controls (CON)	Glucose-tolerant pGDM showed increased liver fat, which suggested that variation in hepatic lipid storage indicates primary and dominant abnormality in this particular group.
Bozkurt et al.; Vienna [35]	Case–control	1H-magnetic resonance spectroscopy	Fatty liver was seen to be increased in GDM	Plasma	3–6 months after delivery over 10 years of observation	This study suggested the indication of excess fat in liver is linked with high risk of deterioration of insulin resistance and manifestation of T2DM and CVS disease.
Sachse et al.; Norway [36]	Case–control	H NMR	Citrate	Maternal urine	visit 1: 8–20 gestational weeks, visit 2: 28 ± 2 weeks, and visit 3: 10–16 weeks postpartum	Study concluded that NMR-based metabolomics can support the changes in monitoring of urinary excretion profile, but it may not be the practical choice for study of GDM.
Garca et al.; Portugal [37]	Case–control	NMR and UPLC-MS	Specific metabolites tested but not specified	Amniotic fluid, blood, and urine	15–25 gestational weeks	The results of the study showed the usefulness of biofluids metabonomics and no significant changes found in between both the groups. Furthermore, follow-up study throughout the pregnancy would give complete metabolic picture.
Diaz et al.; Portugal [38]	Case–control	H NMRS	4- hydroxyphenyl acetate and hippurate were downregulated and choline, glucose, N- methyl nicotinamide, and xylose were upregulated	Urine	14–26	This study demonstrated the maternal urine profile to diagnose prenatal and early prediction of poor outcomes of pregnancy.
Pinto et al.; Portugal [39]	Case–control	NMRS	Pre-diagnosis: valine, proline, urea, pyruvate, 1,5-anhydroglucitol, cholesterol, VLDL, HDL, and LDL Post-diagnosis: alanine, betaine, TMAO, methanol, creatinine, proline, glyceryl, and unsaturated fatty acids	Whole-blood plasma and plasma lipid extracts	2nd and 3rd trimester	Post-diagnosis GDM was classified successfully using 26-resonance plasma biomarker. It also showed possible GDM prediction and diagnosis by the exploiting multivariate profile changes.
Aydemir et al.; Turkey [40]	Case–control	Spectrophotometric method	Downregulation of K167N and polymorphism LOX-1	Blood and plasma	1–18 gestational weeks	The results of the study suggested that in the Turkish group biomarker LOX-1 and K167N polymorphisms might not be involved in susceptibility to GDM and needs further evaluation to check their analysis effects at risk of GDM.
Rottenkolber et al.; Germany [41]	Monocentre cross-sectional analysis	Magnetic resonance spectroscopy	Upregulation of fetuin-A and downregulated insulin sensitivity index	Plasma	At the time of pregnancy and 3–16 months after pregnancy	The conclusion of the study was fetuin-A and leptin signalling were involved in pathogenesis of T2DM.
Pinto et al.; Portugal [42]	Case–control	NMR	3-hydroisovaleric acid, hippurate, choline, creatinine, galactose, lysine, threonine, and phenylacetylglutamine	Urine	2nd and 3rd trimester of pregnancy	12 resonance metabolic signatures at the diagnosis of GDM were identified through this study, furthermore, evaluation of diet therapies and insulin impact enabled to look through metabolic pathways, and identification of side effects were determined.
Jin et al.; China [43]	Case–control	H NMR, biochemical assay, and mRNA extraction	High levels of fasting blood glucose, insulin, mRNA of CD86. Low levels of CX3CLI and CD86.	Blood	N/A	Both the approaches gave information regarding mild GDM, such as amino acid metabolism, fatty acid metabolism, disturbed glucose mechanism, and activated inflammatory response. All these results give insight into underlying mechanisms of mild GDM.

Abbreviations. NMR: Nuclear Magnetic Resonance, HNMRS: Proton Nuclear Magnetic Resonance, IMCL: intramyocellular lipid, HCL: hepatic lipid content, CVS: cardiovascular disease, NMR-UPLC: Nuclear Magnetic Resonance Ultra-performance Liquid Chromatography, NMRS: Nuclear Magnetic Resonance spectroscopy, RNA, LOX-1: lectin-like oxLDL (oxidized low-density lipoprotein) receptor 1, VLDL: very-low-density lipoprotein, HDL: high-density lipoprotein, LDL: low-density lipoprotein, TMAO: trimethylamine N-oxide.

**Table 3:** Summary of GDM Selected Studies General Characteristics and Outcomes of Studies.

Group	Year/Controls	Biomarkers Analysed in Study	Cases	GDM Diagnostic Criteria	Maternal Age	BMI (kg/m²)
Willer et al.; Austria [33]	2003 NGT: 23	IMCL in soleus (IMCL-S) and tibialis anterior muscles (IMCL-T) and leptin system	pGDM: 39, GDM-R: 17 GDM-S: 22	OGTT	GDM: 31.1 ± 0.81 GDM-R: 31.0 ± 1.4 GDM-S: 31.2 ± 0.8 NGT: 30.6 ± 0.9	GDM: 26.4 ± 1.1 GDM-R: 29.8 ± 1.8 GDM-S: 24.9 ± 0.8 NGT: 24.3 ± 0.9
Prikoszovich et al.; Austria [34]	2011 CO: 35	intramyocellular lipids (IMCL) and liver hepatocellular lipids (HCL) and impaired myocellular flux through ATP synthase (fATPase)	PGDM: 37 PGDM IR: 37 PGDM-IS: 39	OGTT	PGDM: 37 ± 5 PGDM-IR: 37 ± 5.9 PGDM-IS: 39 ± 3 CO: 35 ± 4	PGDM: 25.5 ± 3.6 PGDM-IR: 26.5 ± 3 PGDM-IS: 24.2 ± 4.1 CO: 25 ± 2.9
Bozkurt et al.; Vienna [35]	2012 NGT: 29	Determinants of fatty liver and metabolic assessments (IR and free fatty acids)	PGDM-IS: 37 PGDM-IR: 25	OGTT	PGDM-IS: 32.8 ± 4.2 PGDM-IR: 32.5 ± 5.7 NGT: 30.5 ± 5.2	PGDM-IS: 25.4 ± 4.15 PGDM-IR: 30.4 ± 5.4 NGT: 25.4 ± 6.4
Sachse et al.; Norway [36]	2012 NGT:530	leucine, valine, lysine, alanine, tyrosine, formate, histidine, creatine, creatinine N- phenylacetylglycine 3- aminoisobutyrate, 3- hydroxyisovalerate, N- acetylglutamine, dimethylamine, 2- hydroxyisobutyrate trimethylamine N- oxide, glycine, 1- methylnicotinamide, 1,6-anhydroglucose, and 4- hydroxyphenylacetate	GDM: 79	WHO criteria and IADPSG criteria	29.9 ± 4.8	24.6 ± 4.8
Garca et al.; Portugal [37]	2012 20 urine and 23 amniotic samples	Metabonomics	20 urine and 23 amniotic samples	Unknown	>35	N/A
Diaz et al.; Portugal [38]	2013 NGT: 84	Metabolites	GDM: 42	Unknown	N/A	N/A
Pinto et al.; Portugal [39]	2015 NGT:64	Metabolites	Blood plasma: 44 Plasma lipid extracts: 26	IADPSG	Blood: Pre-diag GDM: 30–44 Post-diag GDM: 18–41 Controls: 25–42 Plasma lipid extracts: Pre-diag GDM: 36–42 Post-diag GDM: 18–41 Controls: 28–42	22-26
Aydemir et al.; Turkey [40]	2015 NGT: 120	LOX-1 and K167N	116 pregnant women with GDM	OGTT	GDM: 34.40 ± 5.46 NGT: 35.03 ± 5.46	GDM: 29.4 ± 3.66 NGT: 29.16 ± 1.82
Rottenkolber et al.; Germany [41]	2015 NGT: 51	Fetuin-A, leptin, resistin, adiponectin, and NEFAs	GDM: 96	IADPSG	GDM: 35.9 ± 4 NGT: 35.2 ± 3.9	GDM: 26.3 ± 6.3 NGT: 23.6 ± 4
Pinto et al.; Portugal [42]	2016 Controls: 1 (*n* = 14) Controls: 2 (*n* = 30)	Metabolic profiles	NT: 18 DT: 28 IT: 8	OGTT	N/A	N/A
Jin et al.; China [43]	2017 NGT: 36	Metabolic profiles	GDM: 36	IADPSG	N/A	N/A

Abbreviations. IMCL: intramyocellular lipids, ATP: adenosine triphosphate, IADPSG: International Association of the Diabetes and Pregnancy Study Groups, NGT: normal glucose tolerance, pGDM: previous gestational diabetes mellitus, NEFA: non-esterified fatty acids, OGTT: oral glucose tolerance test.
